# Construction of the Heterostructure of NiPt Truncated Octahedral Nanoparticle/MoS_2_ and Its Interfacial Structure Evolution

**DOI:** 10.3390/nano13111777

**Published:** 2023-05-31

**Authors:** Congyan Mu, Hao Li, Liang Zhou, Huanyu Ye, Rongming Wang, Yinghui Sun

**Affiliations:** Beijing Advanced Innovation Center for Materials Genome Engineering, Beijing Key Laboratory for Magneto−Photoelectrical Composite and Interface Science, School of Mathematics and Physics, University of Science and Technology Beijing, Beijing 100083, China; cy_mu0914@126.com (C.M.); d202110413@xs.ustb.edu.cn (H.L.); b20200364@xs.ustb.edu.cn (L.Z.); b20180341@xs.ustb.edu.cn (H.Y.); rmwang@ustb.edu.cn (R.W.)

**Keywords:** molybdenum disulfide, TEM, metal–semiconductor interaction, surface diffusion

## Abstract

Interfacial atomic configuration plays a vital role in the structural stability and functionality of nanocomposites composed of metal nanoparticles (NPs) and two−dimensional semiconductors. In situ transmission electron microscope (TEM) provides a real−time technique to observe the interface structure at atomic resolution. Herein, we loaded bimetallic NiPt truncated octahedral NPs (TONPs) on MoS_2_ nanosheets and constructed a NiPt TONPs/MoS_2_ heterostructure. The interfacial structure evolution of NiPt TONPs on MoS_2_ was in situ investigated using aberration−corrected TEM. It was observed that some NiPt TONPs exhibited lattice matching with MoS_2_ and displayed remarkable stability under electron beam irradiation. Intriguingly, the rotation of an individual NiPt TONP can be triggered by the electron beam to match the MoS_2_ lattice underneath. Furthermore, the coalescence kinetics of NiPt TONPs can be quantitatively described by the relationship between neck radius (*r*) and time (*t*), expressed as *r^n^ = Kt*. Our work offers a detailed analysis of the lattice alignment relationship of NiPt TONPs on MoS_2_, which may enlighten the design and preparation of stable bimetallic metal NPs/MoS_2_ heterostructures.

## 1. Introduction

Noble metal nanoparticles (NPs) exhibit excellent electrical, optical, and magnetic properties due to the quantum size effect and surface effect [1,2,3]. Typically, Pt NPs play a significant role in various fields, such as catalysis and biomedicine [4,5]. Given the scarcity and high cost of Pt, it is imperative to improve the utilization efficiency of Pt atoms in catalysts. By modulating the exposed crystal facet of Pt NPs, the surface atomic configuration can be changed, even tuning the surface electronic structure of Pt [6,7,8]. Alternatively, the requirement of Pt atoms in catalysts can be significantly reduced by introducing non−noble 3d transition metals (M = Fe, Co, Ni, et al.) to form an alloy with Pt. The synergistic effect between different metal atoms can modify the d−band center position of Pt and enhance its catalytic performance [9,10,11]. Therefore, in the advancement in Pt−based catalysts, attempts have been made to prepare bimetallic MPt alloy NPs with specific control over their shape and exposed facets [12,13,14,15]. In addition, using a proper support can improve the dispersion and availability of Pt NPs. Two−dimensional materials, exemplified by graphene and MoS_2_, possess an atomic−thin layer and high specific surface area. They can be used as highly effective supports for metal NPs and other nanostructures [16,17,18,19]. According to the theoretical calculations and experimental results, S and Pt have a strong electronic coupling which can effectively modulate the catalytic behavior of Pt [20,21,22,23]. Consequently, the construction of a Pt−based bimetallic NPs/MoS_2_ heterostructure holds great potential in reducing the cost of pure Pt NPs. However, the current studies on the metal NPs/MoS_2_ heterostructure primarily focus on the metal NPs with a single component [23,24,25,26,27]. The exploration of the bimetallic NPs/MoS_2_ heterostructure is quite limited. Recently, bimetallic NiPt NPs loaded on MoS_2_ have been used in the sensitive detection of dopamine and uric acid, owing to the good conductivity and catalytic activity of highly dispersed NiPt NPs [28]. The atomic configuration and interaction at the interface greatly affect the performance of metal NPs and even the entire heterostructure [29]. As a result, the precise construction of the interface between NiPt NPs and the MoS_2_ support is very important.

In this work, we prepared a NiPt truncated octahedral NPs (NiPt TONPs)/MoS_2_ heterostructure by ultrasonic loading. Through in situ TEM observations, we comprehensively explored the structural evolution at the interface. We found that certain ultrasonically loaded NiPt TONPs reached a lattice match with the MoS_2_ substrate, exhibiting remarkable stability under electron beam irradiation. The NiPt TONP that did not reach the lattice match with MoS_2_ rotated to epitaxially align under electron beam irradiation. Additionally, the adjacent NiPt TONPs coalesced on MoS_2_ with kinetics showing a slow and then a fast rate under prolonged irradiation. The NP rotated to align the lattice orientation at the interface of adjacent NPs during coalescence. Our work provides a detailed analysis of the structural evolution of bimetallic NiPt TONPs on MoS_2_, which is helpful for the design, preparation, and stability study of a bimetallic metal NPs/MoS_2_ heterostructure with desired properties.

## 2. Materials and Methods

### 2.1. Chemicals and Materials

All reagents were of analytical grade and used as purchased, without further purification. Platinum acetylacetonate (Pt(acac)_2_) and nickel acetylacetonate (Ni(acac)_2_) were purchased from Alfa Aesar. Polyvinylpyrrolidone (PVP) and molybdenum disulfide nanosheets (MoS_2_, 100 nm) were purchased from Macklin. N, N−dimethylformamide (DMF) was purchased from Beijing Jingchun Reagent. Ethanol and acetone were purchased from Sinopharm Chemical Reagent Co., Ltd., Shanghai, China.

### 2.2. Synthesis of NiPt TONPs

The solvothermal method is an important approach for the preparation of various nanomaterials and their composites [30,31]. NiPt TONPs were synthesized using a solvothermal method according to a published procedure [31]. In a typical synthesis, 28 mg of Pt(acac)_2_, 14 mg of Ni(acac)_2_, and 70 mg PVP were dissolved in 20 mL DMF to form a homogeneous solution under sonication for 0.5 h. Then, the solution was transferred into a 25 mL Teflon−lined stainless−steel autoclave, heated to 180 °C, and kept for 12 h before it was cooled down to room temperature. The product was further washed with ethanol and acetone several times, and finally dissolved in ethanol.

### 2.3. Preparation of NiPt TONPs/MoS_2_ Heterostructure

In a typical preparation, 2 mg of MoS_2_ nanosheets was dissolved in 10 mL ethanol in a vial and ultrasonicated. The ethanol solution of NiPt TONPs was added dropwise to a vial with a pipette. Then, the mixture was sonicated for 2 h. The sonication temperature was controlled at room temperature to prevent the coalescence of NiPt TONPs.

### 2.4. Characterizations

The morphology and structure of NiPt TONPs and the NiPt TONPs/MoS_2_ heterostructure were characterized by a field−emission TEM (JEOL JEM−2200FS, operated at 200 kV) and an image−aberration−corrected TEM (FEI Titan ETEM G2, operated at 300 kV). The samples ultrasonically dispersed in ethanol were dropped by a pipette on a Cu TEM grid coated with porous carbon. X−ray photoelectron spectroscopy (XPS) was performed with an X−ray energy spectrometer (PHI 5000 VersaProbe III) with monochromatic Al K_α_ X−ray. The value of binding energy was calibrated by setting that of the adventitious carbon C 1s to 284.6 eV.

## 3. Results and Discussion

**Figure 1:**
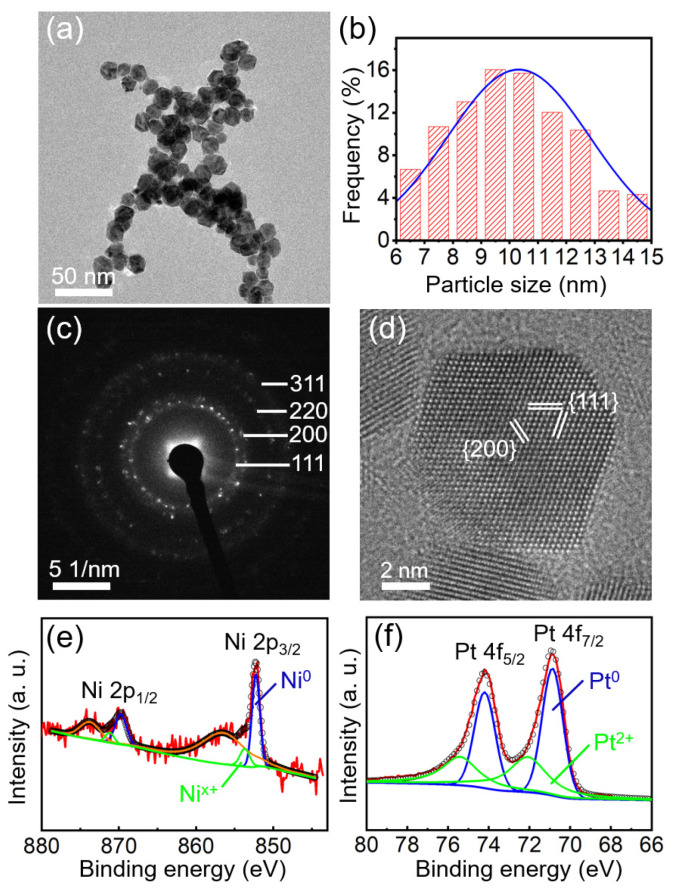
(**a**) Low−magnification TEM image of NiPt TONPs; the histogram of size distribution (**b**); the corresponding SAED pattern of NiPt TONPs (**c**); (**d**) HRTEM image of a typical NiPt TONP; and high−resolution XPS spectra of (**e**) Ni 2p and (**f**) Pt 4f for NiPt TONPs, respectively.

The microstructure of the as−prepared NiPt NPs characterized by TEM is shown in Figure 1a, which reveals their truncated octahedral (TO) morphology. The statistical average of NP sizes is about 10.3 nm (Figure 1b). The selected area’s electron diffraction (SAED) pattern in Figure 1c reveals the crystallization of NiPt TONPs. Clear concentric rings can be observed with the radii measured as 0.218, 0.190, 0.134, and 0.117 nm, respectively. They can be indexed to the {111}, {200}, {220}, and {311} crystal planes of NiPt face−centered cubic (fcc) structure, respectively. This result is consistent with previous reports [31,32]. A well−defined crystal structure of an individual NiPt TONP was characterized by high−resolution TEM (HRTEM), as shown in Figure 1d. The d−spacings of 0.217 and 0.189 nm correspond to the {111} and {200} crystal planes of Pt fcc structure, respectively. Notably, the coherent extension of the lattice fringes throughout the entire NP confirms its single−crystal structure. 

The XPS spectra of Ni (Figure 1e) and Pt (Figure 1f) were calibrated using the C 1s peak at a binding energy of 284.6 eV as a reference. Three pairs of double peaks can be fitted in Figure 1e: Ni^0^, Ni^x+^, and the Ni−satellite peaks [15]. The peaks located at 869.73 and 852.24 eV correspond to Ni 2p_1/2_ and Ni 2p_3/2_, respectively. According to the XPS handbook, the Ni^x+^ peaks, located at 871.24 and 853.75 eV, can be assigned to oxidized Ni (Ni^2+^) [33]. Similarly, for the Pt 4f spectrum shown in Figure 1f, two pairs of double peaks can be fitted, which are attributed to metallic Pt^0^ peak (74.22 and 70.89 eV), and the Pt^2+^ peak (75.48 and 72.15 eV) in PtO or Pt(OH)_2_ [15,31,34]. It is noted that the XPS signal of Ni is weaker than that of Pt, presumably suggesting the lower Ni content or the Ni−scarce surface of the NPs. After all, as a surface analysis technique, XPS can only probe the elemental signals from the top few nanometers of the analyzed samples.

**Figure 2:**
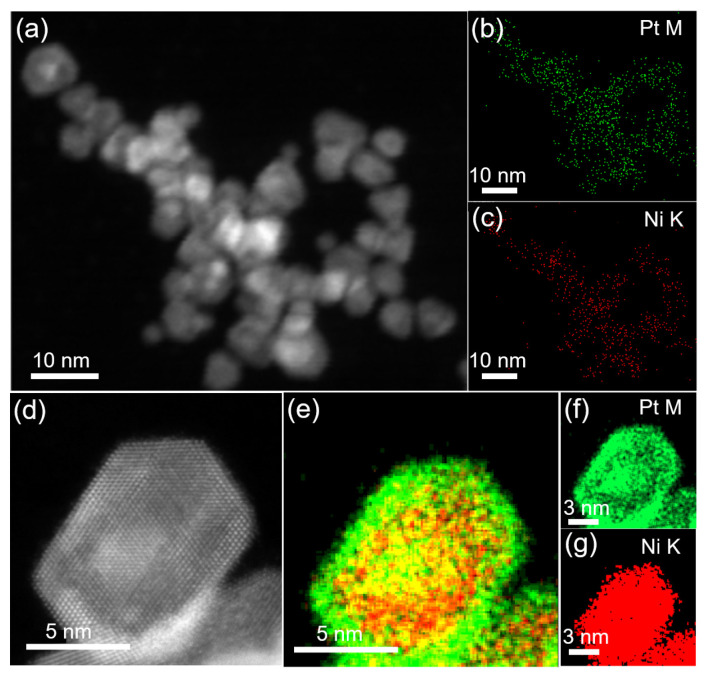
(**a**) STEM image of NiPt TONPs; corresponding EDS elemental mappings of Pt (**b**) and Ni (**c**); (**d**) representative HAADF−STEM image of a typical NiPt TONP; and (**e**–**g**) corresponding Pt and Ni elemental mappings.

To study the content and distribution of Ni and Pt elements in NiPt TONPs, scanning TEM (STEM) and X−ray energy spectroscopy (EDS) were used. Figure S1 shows the composition investigated by EDS. Except for the characteristic peaks of Cu elements in the Cu TEM grid, only peaks of Ni and Pt elements were observed. It is reasonable to deduce that the synthesized products only consist of Ni and Pt elements. The STEM image in Figure 2a shows the three−dimensional structure of the NPs. The distribution of Pt and Ni elements are revealed in Figure 2b,c, respectively, showing a uniform distribution of Pt and Ni with the atomic percentage of 63.6% for Pt and 36.4% for Ni. This indicates a relatively low content of Ni in the NPs, which is consistent with the XPS results above. High−angle annular dark−field (HAADF) STEM can further determine the elemental composition of an individual NP. Figure 2d shows a typical HAADF−STEM image of an individual NiPt TONP, and the corresponding Pt and Ni elemental mappings are shown in Figure 2e–g. They clearly demonstrate that Ni is mainly distributed in the central region of the NP, while Pt is distributed throughout the whole NP. The surface of the NiPt TONP is Pt−rich and Ni−scarce, together with the low content of Ni in the atomic percentage, which result in the weak XPS signal of Ni.

**Figure 3:**
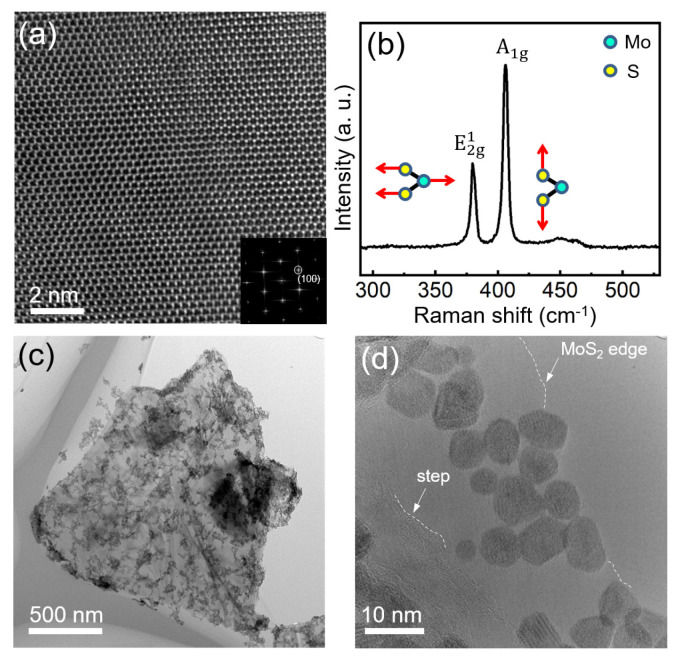
(**a**) HRTEM image of MoS_2_, the inset is the corresponding FFT image of (**a**); (**b**) Raman spectra of MoS_2_ nanosheets; and (**c**,**d**) low- and high-magnification TEM images of the NiPt TONPs decorated on MoS_2_. The white dashed curves indicate the edge of MoS_2_ nanosheet or steps of different layers.

The NiPt TONPs/MoS_2_ heterostructure was prepared by loading NiPt TONPs on MoS_2_ nanosheets at room temperature. Figure 3a presents a typical HRTEM image of the pristine MoS_2_ nanosheet before decoration. The image reveals continuous crystal lattice fringes, while the inset shows the corresponding fast Fourier transform (FFT) pattern, confirming the excellent crystallinity. Raman spectroscopy excited by a 532 nm laser in the environmental condition was employed to study the structural vibration modes of MoS_2_, as shown in Figure 3b. The typical $E_{2g}^{1}$ and $A_{1g}$ peaks at about 380.0 and 405.9 cm^−1^ belong to the 2H crystalline phase of MoS_2_. It is well known that the $E_{2g}^{1}$ mode results from the in−plane opposite vibration of the two S atoms relative to the Mo atom between them. The $A_{1g}$ mode originates from the out−of−plane vibration of only the S atoms in opposite directions. The separation between the two Raman modes is about 25.9 cm^−1^ (extracted by a Lorentzian peak fitting), which matches the result of few−layer MoS_2_ [35]. Low− and high−magnification TEM images of the NiPt TONPs decorated on MoS_2_ nanosheets are shown in Figure 3c,d, indicating a good loading amount of the NPs. NiPt TONPs are mostly attached to the edge of MoS_2_ nanosheet or steps of different layers.

The dynamic structure evolution of the NiPt TONPs/MoS_2_ heterostructure under electron beam irradiation was observed using in situ TEM. Figure S2(a_1_–d_1_) show the HRTEM images of a typical NiPt TONP on MoS_2_ irradiated by the electron beam at 0, 47, 85, and 118 s, respectively. The average electron beam dose rate was around 1.66 × 10^6^ e^−^/nm^2^•s^−1^ at 300 kV. As time elapsed, the NP on MoS_2_ was quite stable and its crystallinity was promoted. The corresponding FFT images of Figure S2(a_1_–d_1_) are shown in Figure S2(a_2_–d_2_), respectively. Clear FFT points from MoS_2_ can be obtained in Figure S2(a_2_), marked by the yellow hexagonal frame, which was not a regular hexagon because the crystal zone axis of MoS_2_ was not hexagonal [001]. The measured d−spacings are 0.224, 0.225, and 0.260 nm at angles of 50.0°, 64.8°, and 65.2°, respectively. These are consistent with the crystal zone axis [331] of 2H−MoS_2_, and the crystal plane is shown in Figure S2(a_2_). The FFT point of the NP was marked by the green circle, and the d−spacing was measured as 0.225 nm, which is calibrated as the (111) crystal plane of NiPt TONP. Notably, using the digital dark−field method by filtering the FFT point marked by the green circle in Figure S2(a_2_–d_2_), the epitaxial alignment in this direction can be extracted. The corresponding inverse FFT patterns in Figure S2(a_3_–d_3_) contain the lattice from both MoS_2_ and the NiPt TONP. These patterns reveal a gradual clarified surface and an improved crystallinity of the NP under electron beam irradiation. The crystallographic orientation relationship is (111)_NiPt_//( $\bar{1}$03)_MoS2_, as marked by the white dashed frames in Figure S2(a_2_−d_2_). The result indicates that the NiPt TONP with lattice match to MoS_2_ was very stable during the ultrasonic assembly process.

**Figure 4:**
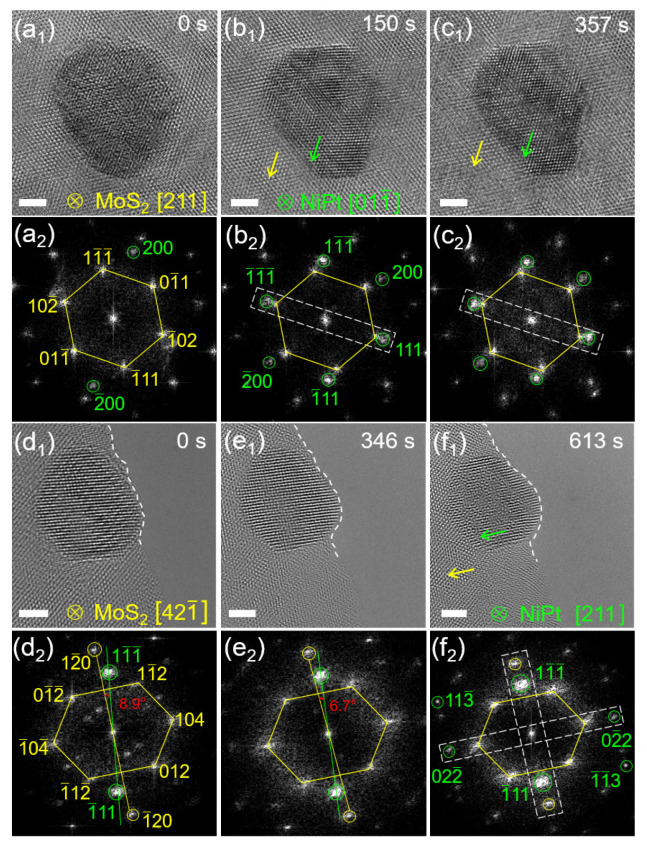
(**a_1_**–**c_1_**) Time series HRTEM images of a NiPt TONP on MoS_2_ at 0, 150, and 357 s; (**a_2_**–**c_2_**) FFT patterns corresponding to (**a_1_**–**c_1_**); (**d_1_**–**f_1_**) time series HRTEM images of another NiPt TONP on MoS_2_ at 0, 346, and 613 s; and (**d_2_**–**f_2_**) FFT patterns corresponding to (**d_1_**–**f_1_**). The scale bars are all 2 nm.

To investigate whether all NiPt TONPs loaded on MoS_2_ are stable, other NPs were selected for observation and analysis. Figure 4(a_1–_c_1_) show the HRTEM images of another typical NiPt TONP on MoS_2_ irradiated by the electron beam at 0, 150, and 357 s, respectively. As time elapsed, the NP on MoS_2_ revealed a clearer TO shape and the crystallinity was improved. The corresponding FFT patterns in Figure 4(a_1_–c_1_) are shown in Figure 4(a_2_–c_2_), respectively. Clear FFT points from MoS_2_ can be obtained in Figure 4(a_2_), marked by the yellow hexagonal frame, which was not a regular hexagon because the MoS_2_ crystal zone axis was not hexagonal [001]. The measured d−spacings are 0.267, 0.263, and 0.252 nm at angles of 63.9°, 57.6°, and 58.5°, respectively. These are consistent with the crystal zone axis [211] of 2H−MoS_2_, with the crystal plane shown in Figure 4(a_2_). The green circles in Figure 4(a_2_–c_2_) indicate the FFT points from the NiPt TONP. In Figure 4(b_2_), the measured d−spacings of 0.220, 0.221, and 0.193 nm at angles of 70.1°, 54.5°, and 55.4°, respectively, can be identified as the [01$\bar{1}$] crystal zone axis of NiPt, with the crystal plane shown in Figure 4(b_2_). By comparing the FFT points obtained from the (200) crystal plane of the NP in Figure 4(a_2_,b_2_), a distinct rotation of 36° over time can be observed. This rotation indicates that the NP undergoes a reorientation process under electron beam irradiation to achieve lattice match with MoS_2_. The (111) crystal plane of NP is epitaxial to the ($\bar{1}$02) crystal plane of MoS_2_, as indicated by the white dashed frames in Figure 4(b_2_). This crystalline orientation was observed to be maintained over time, indicating that a stable orientation of the NP on MoS_2_ was obtained. The crystallographic orientation relationship is (111)_NiPt_//($\bar{1}$02)_MoS2_, [01$\bar{1}$]_NiPt_//[211]_MoS2_.

To investigate whether the rotation behavior of NiPt TONP on MoS_2_ is universal, other NPs were selected for TEM observation. Figure 4(d_1–_4f_1_) show the HRTEM images of another typical NiPt TONP on MoS_2_ irradiated by the electron beam at 0, 346, and 613 s, respectively. Figure 4(d_2_–f_2_) show the corresponding FFT patterns in Figure 4(d_1_–f_1_), respectively. Clear FFT points from MoS_2_ can be seen in Figure 4(d_2_), marked by the yellow hexagonal frame, which also reflects that the MoS_2_ crystal zone axis was not hexagonal [001]. The measured d−spacings are 0.250, 0.250, and 0.212 nm at angles of 74.5°, 53.1°, and 52.4°, respectively. These are consistent with the crystal zone axis [331] of 2H−MoS_2_, with the crystal plane shown in Figure 4(d_2_). Clear FFT points from the NP can be obtained in Figure 4(f_2_), marked by green circles. The measured d−spacings are 0.112, 0.226, and 0.133 nm at angles of 58.4°, 91.6°, and 30.0°, respectively, which can be identified as the [211] crystal zone axis of NiPt, and the crystal plane is shown in Figure 4(f_2_). Figure 4(d_2_–4f_2_) indicate the gradual rotation of the (1$\bar{1}$ $\bar{1}$) crystal plane of the NP. The included angle between the (1$\bar{1}$ $\bar{1}$) crystal plane of the NP and the (1$\bar{2}$0) crystal plane of MoS_2_ is 8.9° in Figure 4(d_2_), which changes to 6.7° in Figure 4(e_2_) and 0° in Figure 4(f_2_). This means that the NP rotated to match the MoS_2_ lattice plane, as indicated by the white dashed frames in Figure 4(f_2_). The crystallographic orientation relationship is (1$\bar{1}$ $\bar{1}$)_NiPt_//(1$\bar{2}$0)_MoS2_, (0$\bar{2}$2)_NiPt_//(104)_MoS2_, [211]_NiPt_//[42$\bar{1}$]_MoS2_. Therefore, the decorated NiPt TONPs on MoS_2_ rotates to match the MoS_2_ lattice fringe when irradiated by the electron beam.

Besides the rotation of NiPt TONP on MoS_2_, the surface atoms of NiPt TONPs may be reorganized under electron beam irradiation. Figure S3(a_1_−d_1_) show the HRTEM images of another NiPt TONP on MoS_2_ irradiated by the electron beam at 0, 101, 561, and 689 s, respectively. As time elapsed, the shape of the NP changed, probably due to the diffusion of surface atoms on the exposed crystal facets. Figure S3(a_2_–d_2_) show the corresponding FFT patterns in Figure S3(a_1_–d_1_), respectively. Clear FFT points from MoS_2_ can be seen in Figure S3(a_2_), marked by the yellow hexagonal frame. The measured d−spacings are 0.256, 0.266, and 0.252 nm at angles of 62.2°, 61.6°, and 56.2°, respectively. These are consistent with the crystal zone axis [221] of 2H−MoS_2_, and the crystal plane is shown in Figure S3(a_2_). Clear FFT points from the NP can be seen in Figure S3(b_2_), marked by the green circles. The measured d−spacing are 0.189, 0.114, and 0.113 nm at angles of 73.8°, 32.4°, and 73.8°, respectively, which can be identified as the [031] crystal zone axis of NiPt, and the crystal plane is shown in Figure S3(b_2_). It can be recognized that the (1$\bar{1}$3) crystal plane of the NP and the (0$\bar{2}$4) crystal plane of MoS_2_ are not aligned, and the (111) crystal lattice fringes disappeared and shifted to the [031] crystal band axis of the NP. In Figure S3(c_2_), the FFT points from (1$\bar{1}$3) crystal plane of the NP and (0$\bar{2}$4) crystal plane of MoS_2_ aligned and maintained this epitaxial alignment over time, as indicated by the white dashed frames. The matching relationship is (1$\bar{1}$ 3)_NiPt_//(0$\bar{2}$4)_MoS2_, [031]_NiPt_//[221]_MoS2_.

**Figure 5:**
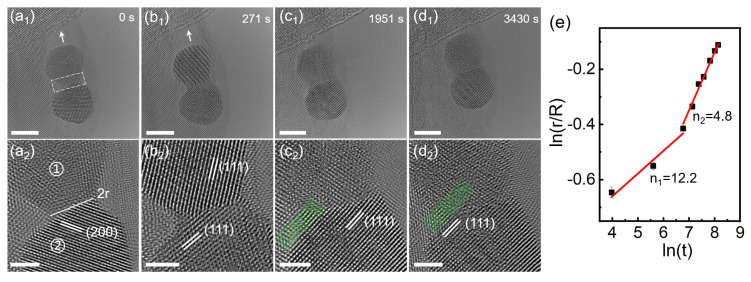
(**a_1_**–**d_1_**) Time series TEM images of the coalescence behavior of two adjacent NiPt TONPs on MoS_2_ at 0, 271, 1951, and 3430 s. The scale bars are 5 nm; (**a_2_**–**d_2_**) time series HRTEM images of the corresponding neck area in (**a_1_**–**d_1_**). The scale bars are 2 nm; (**e**) the relationship between the neck radius and the natural logarithm of time, which is fitted using a least-squares approximation. The error bars in the graph represent the standard deviation derived from five measurements of the neck radius taken at the corresponding moments.

In addition to the rotation of an individual NiPt TONP and the surface atom diffusion, the coalescence of two adjacent NiPt TONPs on MoS_2_ was also observed under electron beam irradiation. Figure 5(a_1_–d_1_) show the TEM images of two adjacent NiPt TONPs irradiated by the electron beam at 0, 271, 1951, and 3430 s, respectively. As time elapsed, the shape of the NPs gradually became rounded, and they approached the step of MoS_2_, as indicated by the white arrows. Figure 5(a_2_–d_2_) show the intercepted HRTEM image at the neck of the two NPs for the detailed analysis of the coalescence process. The upper NP was labeled “1” and the lower one was labeled “2”. Once the two adjacent NPs contacted each other, the surface atom diffusion was accelerated to form a neck, marked by the white dashed frames in Figure 5(a_1_). The neck width (2*r*) gradually increased with the irradiation of the electron beam, which was measured as 3.3, 4.1, 5.7, and 6.4 nm in Figure 5(a_2_–d_2_), respectively. In Figure 5(a_2_), the d−spacing of NP “2” is 0.192 nm, labeled the (200) crystal plane of NiPt, while the crystal lattice of NP “1” cannot be seen clearly. In Figure 5(b_2_), the d−spacing of NP “1” is 0.221 nm, labeled the (111) crystal plane of NiPt. In Figure 5(b_2_–d_2_), the d−spacing of NP “2” is 0.223 nm, labeled the (111) crystal plane of NiPt. Through structure evolution under electron beam irradiation, the crystal lattice fringe became continuous at the interface of the two NPs, as shown in the green shadowed area in Figure 5(c_2_,d_2_). 

The coalescence process of NPs can be quantitatively described by an expression linking the neck radius (*r*) and time (*t*) as *r^n^ = Kt*. In this equation, *n* signifies the characteristic variable related to the mass transport mechanism, while *K* is a constant depending on the intrinsic factors, such as the average radius of the NPs, atomic volume, temperature, surface energy, and the diffusivity of the material [36,37]. The power law, derived from the classical continuum model, has been widely used to describe the dynamic coalescence phenomena of NPs [38,39,40]. Utilizing the obtained experimental data, we plotted the evolution of *r* with the natural logarithmic of *t*, which were fitted by using a least-squares approximation (Figure 5e). The coalescence ranged from the *r* at the beginning of contact (*r* = 1.67 nm) to the initial value of the NP radius (*R* = 3.56 nm), showing two linear dependencies of the *r* on the natural logarithm of *t* with slope (1/*n*). The slope was 0.08 and *n* ≈ 12.2 for the first stage, while it was 0.21 and *n* ≈ 4.8 for the second stage. The larger value of *n* in the first stage may be due to the relatively large NP size and the more resistant sintering of the Pt−rich surface [41]. The smaller *n* in the second stage indicates a faster coalescence process, which may be attributed to the highly mobile atoms on the irradiated NPs surface.

Figure S4a–c show TEM images of the coalescence of another pair of adjacent NiPt TONPs at 0, 166, and 267 s, respectively. The d−spacing along the arrow direction for NP “1” and “2” was measured to be 0.223 nm, marked as the (111) crystal plane of NP. In Figure S4a, the angle between the (111) crystal plane direction and the vertical direction of NP “1” was 56°, and the NP “2” was 45°, which shows that the (111) crystal plane of the two NPs had an angle of 11° at the initial moment. Under the electron beam irradiation, NP “2” underwent a rotation and achieved the same lattice orientation at the interface of the two NPs, as shown in Figure S4b,c.

## 4. Conclusions

In summary, we successfully prepared a NiPt TONPs/MoS_2_ heterostructure by loading NiPt TONPs on MoS_2_ nanosheets. The interfacial structural evolution of the heterostructure under electron beam irradiation was analyzed in detail. In an individual NiPt TONP, Ni and Pt elements segregated. We found that some of the ultrasonically loaded NiPt TONPs reached a lattice match with MoS_2_ which was quite stable under electron beam irradiation. The NiPt TONP that did not achieve the lattice match with MoS_2_ rotated to epitaxially align under electron beam irradiation. This process typically takes about 150 to 600 s. It was consistently observed that when NiPt TONP stabilized on MoS_2_, a lattice matching relationship between the two was present in specific facets. We found some typical crystallographic orientation relationships of NiPt TONPs on MoS_2_: (111)_NiPt_//($\bar{1}$03)_MoS2_; (111)_NiPt_//( $\bar{1}$02)_MoS2_, [01$\bar{1}$]_NiPt_//[211]_MoS2_; (1$\bar{1}$$\bar{1}$)_NiPt_//(1$\bar{2}$0)_MoS2_, (0$\bar{2}$2)_NiPt_//(104)_MoS2_, [211]_NiPt_//[42$\bar{1}$]_MoS2_; or (1$\bar{1}$3)_NiPt_//(0$\bar{2}$4)_MoS2_, [031]_NiPt_//[221]_MoS2_. In addition, the adjacent NiPt TONPs coalesced on MoS_2_ with kinetics showing a slow and then fast rate under prolonged irradiation, and the NP rotated to align the lattice orientation at the interface of adjacent NPs during coalescence. Our study presents a comprehensive analysis of the structural evolution of bimetallic NiPt TONPs on MoS_2_, offering valuable insights for the design, preparation, and stability study of a bimetallic metal NPs/MoS_2_ heterostructure with tailored properties.

## Data Availability

Not applicable.

## Supplementary Materials

The following supporting information can be downloaded at: https://www.mdpi.com/article/10.3390/nano13111777/s1, Figure S1: EDS spectrum of NiPt TONPs; Figure S2: (a_1_–d_1_) Time series HRTEM images of a NiPt TONP on MoS_2_ at 0, 47, 85, and 118 s; (a_2_–d_2_) FFT patterns corresponding to (a_1_–d_1_); (a_3_–d_3_) Inverse FFT patterns corresponding to the green circles in (a_2_–d_2_); Figure S3: (a_1_–d_1_) Time series HRTEM images of a NiPt TONP on MoS_2_ at 0, 101, 561, and 689 s; (a_2_–d_2_) FFT patterns corresponding to (a_1_–d_1_); Figure S4: (a–c) Time series TEM images of the coalescence of two adjacent NiPt TONPs on MoS_2_ at 0, 166, and 267 s.
