# Supplementary material for: Construction of the Heterostructure of NiPt Truncated Octahedral Nanoparticle/MoS2 and Its Interfacial Structure Evolution
Source: Nanomaterials (Basel). 2023 May 31;13(11):1777. doi: 10.3390/nano13111777 (PMC10255085; doi:10.3390/nano13111777)
Supplement: Supplementary file 1 [file nanomaterials-13-01777-s001.zip › nanomaterials-2420714-supplementary.pdf]

nanomaterials

Supplementary Materials

Construction of the heterostructure of NiPt truncated octahedral nanoparticle/MoS_2 and its interfacial structure evolution

Congyan Mu, Hao Li, Liang Zhou, Huanyu Ye, Rongming Wang and Yinghui Sun*

Beijing Advanced Innovation Center for Materials Genome Engineering, Beijing Key Laboratory for Magneto−Photoelectrical Composite and Interface Science, School of Mathematics and Physics, University of Science and Technology Beijing, Beijing 100083, China; cy_mu0914@126.com (C.M.); D202110413@xs.ustb.edu.cn (H.L.); b20200364@xs.ustb.edu.cn (L.Z.); b20180341@xs.ustb.edu.cn (H.Y.); rmwang@ustb.edu.cn (R.W.)

* Correspondence: yhsun@ustb.edu.cn (Y.S.)

1. EDS spectrum of NiPt TONPs

The NiPt TONPs prepared by the solvothermal method were loaded onto MoS_2 nanosheets by the ultrasonic assembly. Then the product was ultrasonically dispersed in ethanol and dropped by a pipette on a Cu TEM grid coated with porous carbon film. Figure S1 shows the composition investigated by EDS. Except for the characteristic peaks of Cu elements in the Cu TEM grid, only the peaks of Ni and Pt elements were observed. It is reasonable to conclude that the synthesized products only consist of Ni and Pt elements.

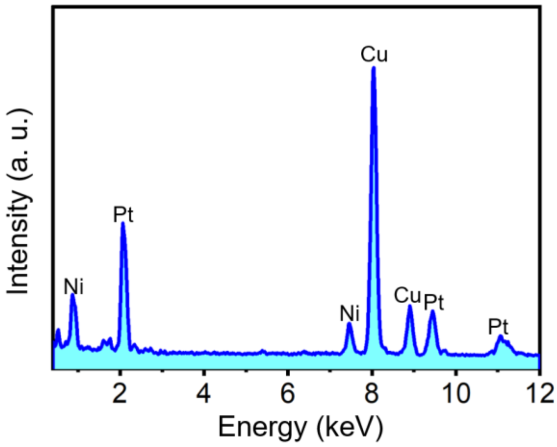

Figure S1. EDS spectrum of NiPt TONPs.

Copyright: © 2023 by the authors. Licensee MDPI, Basel, Switzerland. This article is an open access article distributed under the terms and conditions of the Creative Commons Attribution (CC BY) license (https://creativecommons.org/licenses/by/4.0/).

2. Structure evolution of a NiPt TONP on MoS_2 under electron beam irradiation

Figure S2 shows the dynamic structure evolution of a NiPt TONP on MoS_2 under electron beam irradiation. With time elapse, the NP on MoS_2 was quite stable and the crystallinity was promoted. By measuring the d−spacings and the corresponding angles in Fig. S2a_2, it can be calibrated as the crystal zone axis [331] of 2H−MoS_2 and (111) crystal plane of NiPt. As indicated by the white dashed frames in Figs. S2a_2−S2d_2, the NP and MoS_2 have a crystallographic orientation relationship of $(111)_{NiPt}//(\bar{1}03)_{MoS_2}$. The result indicates that the NiPt TONP with lattice match to MoS_2 was very stable during the ultrasonic assembly process.

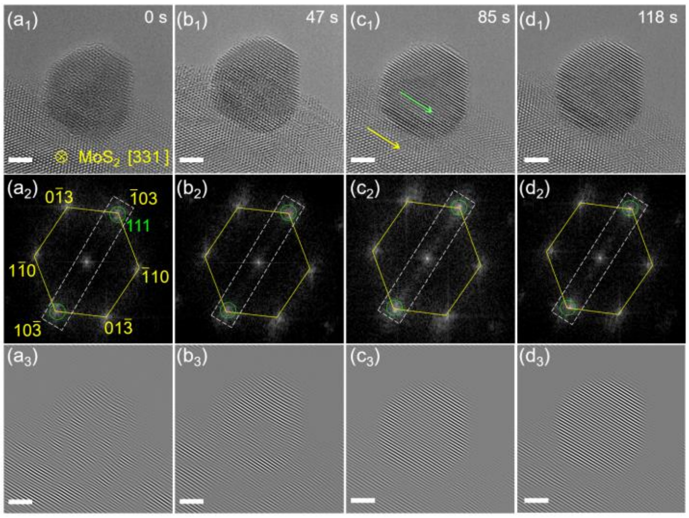

Figure S2. (a_1–d_1) Time series HRTEM images of a NiPt TONP on MoS_2 at 0, 47, 85, and 118 s; (a_2–d_2) FFT patterns corresponding to (a_1–d_1); (a_3–d_3) Inverse FFT patterns corresponding to the green circles in (a_2–d_2). The scale bars are all 2 nm.

3. Structure evolution of another NiPt TONP on MoS_2 under electron beam irradiation

Figure S3 shows the dynamic structure evolution of a NiPt TONP on MoS_2 under electron beam irradiation. With time elapse, the shape of the NP was changed, probably due to the diffusion of surface atoms on the exposed crystal facets. According to the d–spacings and the corresponding angle in Fig. S3b2, it can be calibrated as the crystal zone axis [221] of 2H–MoS_2 and [031] of NiPt. From Figs. S3a2–S3d2, it can be seen that the NP rotated under electron beam irradiation to achieve a lattice matching relationship with MoS_2 of $(1\bar{1}3)_{NiPt}//(0\bar{2}4)_{MoS_2}$, $[031]_{NiPt}//[221]_{MoS_2}$.

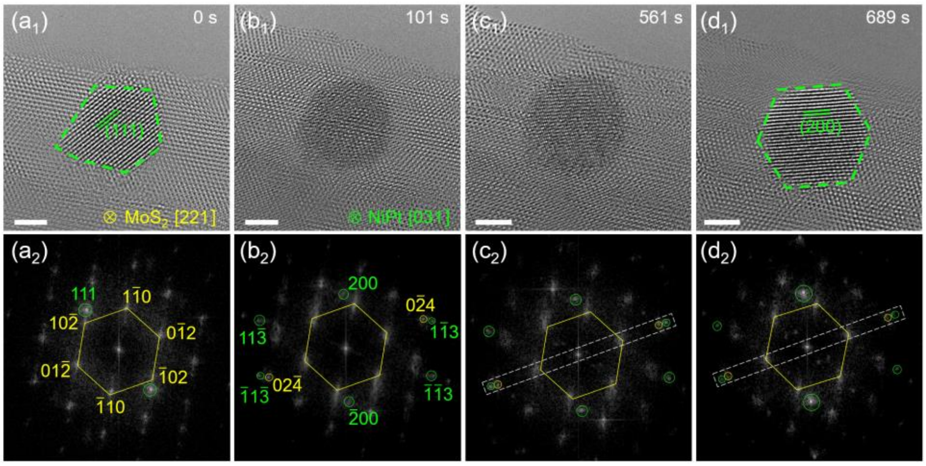

Figure S3. (a_1–d_1) Time series HRTEM images of a NiPt TONP on MoS_2 at 0, 101, 561, and 689 s; (a_2–d_2) FFT patterns corresponding to (a_1–d_1). The scale bars are all 2 nm.

4. Coalescence of two adjacent NiPt TONPs on MoS_2 under electron beam irradiation

Figure S4 shows the coalescence behavior of two adjacent NiPt TONPs on MoS_2 irradiated by the electron beam. With time elapse, the NP "2" undergoes a rotation and achieves the same lattice orientation at the interface of the two NPs.

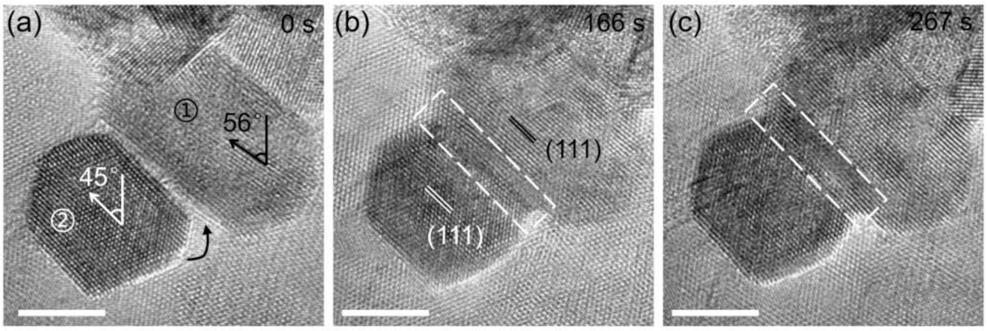

Figure S4. (a–c) Time−series TEM images of the coalescence of two adjacent NiPt TONPs on MoS_2 at 0, 166, and 267 s. The scale bars are all 5 nm.

Disclaimer/Publisher's Note: The statements, opinions and data contained in all publications are solely those of the individual author(s) and contributor(s) and not of MDPI and/or the editor(s). MDPI and/or the editor(s) disclaim responsibility for any injury to people or property resulting from any ideas, methods, instructions or products referred to in the content.
